# Social Media Outrage in Response to a School-Based Substance Use Survey: Qualitative Analysis

**DOI:** 10.2196/15298

**Published:** 2019-09-12

**Authors:** Ruth Ann Gassman, Tapati Dutta, Jon Agley, Wasantha Jayawardene, Mikyoung Jun

**Affiliations:** 1 Prevention Insights, Institute for Research on Addictive Behavior School of Public Health Indiana University Bloomington Bloomington, IN United States; 2 Rural Center for AIDS/STD Prevention School of Public Health Indiana University Bloomington Bloomington, IN United States

**Keywords:** social media, ATOD, survey, firestorm, digital

## Abstract

**Background:**

School-based alcohol, tobacco, and other drug use (ATOD) surveys are a common epidemiological means of understanding youth risk behaviors. They can be used to monitor national trends and provide data, in aggregate, to schools, communities, and states for the purposes of funding allocation, prevention programming, and other supportive infrastructure. However, such surveys sometimes are targeted by public criticism, and even legal action, often in response to a lack of perceived appropriateness. The ubiquity of social media has added the risk of potential online firestorms, or digital outrage events, to the hazards to be considered when administering such a survey. Little research has investigated the influence of online firestorms on public health survey administration, and no research has analyzed the content of such an occurrence. Analyzing this content will facilitate insights as to how practitioners can minimize the risk of generating outrage when conducting such surveys.

**Objective:**

This study aimed to identify common themes within social media comments comprising an online firestorm that erupted in response to a school-based ATOD survey in order to inform risk-reduction strategies.

**Methods:**

Data were collected by archiving all public comments made in response to a news study about a school-based ATOD survey that was featured on a common social networking platform. Using the general inductive approach and elements of thematic analysis, two researchers followed a multi-step protocol to clean, categorize, and consolidate data, generating codes for all 207 responses.

**Results:**

In total, 133 comments were coded as oppositional to the survey and 74 were coded as supportive. Among the former, comments tended to reflect government-related concerns, conspiratorial or irrational thinking, issues of parental autonomy and privacy, fear of child protective services or police, issues with survey mechanisms, and reasoned disagreement. Among the latter, responses showed that posters perceived the ability to prevent abuse and neglect and support holistic health, surmised that opponents were hiding something, expressed reasoned support, or made factual statements about the survey. Consistent with research on moral outrage and digital firestorms, few comments (<10%) contained factual information about the survey; nearly half of the comments, both supportive and oppositional, were coded in categories that presupposed misinformation.

**Conclusions:**

The components of even a small online firestorm targeting a school-based ATOD survey are nuanced and complex. It is likely impossible to be fully insulated against the risk of outrage in response to this type of public health work; however, careful articulation of procedures, anticipating specific concerns, and two-way community-based interaction may reduce risk.

## Introduction

### School-Based Public Health Surveys

School-based alcohol, tobacco, and other drug (ATOD) surveys are internationally recognized as one of the most important ways of understanding ATOD use and related risk behaviors among young people [1]. In the United States, such surveys often include questions on attitudes (eg, perceived harm of substance use) and behaviors (eg, bullying); they are also used to monitor national trends [2] and provide aggregated information to school districts, states, and communities [3]. Administration of these surveys continues to be important, especially in the midst of a national addictions crisis that affects youth and adolescents [4] and the continually changing patterns of substance use and risk behaviors in this population, such as the recent surge in e-cigarette use [5]. Survey data and reports are also valuable in supporting continued efforts to design effective state and local education standards for substance use prevention [6] and evidence-based prevention strategies [7].

Though these surveys are designed for public health surveillance and prevention, there is a risk that they will be misunderstood as experimental research being conducted on youth, which occupies a more controversial space in public discourse [8]. Such misunderstandings, in conjunction with potentially contentious questionnaire topics like sexual health and substance use, have led to federal lawsuits against schools for administering surveys; however, two notable federal appellate cases had been resolved prior to 2007 in favor of the school districts [9]. Nonetheless, public health surveys continue to be attacked in traditional and online media [10-12], although such instances are rarely documented in academic literature. At times, clusters of incidents have also been captured in blog posts advocating for a specific position on school-based surveillance surveys [13]. School responses to such events, documented in these stories and posts [10-13], have ranged from a simple apology to cessation of survey administration.

### Nature and Impact of Public Controversy

Although little research has focused on the outcomes of public controversy on school-based surveys, such negative exposure is known to have a “chilling effect” on public health scholars in general, leading to self-censorship, modification of agendas, and even career changes [14]. Controversy can also serve as an informal social control on the types of information that is *acceptable* to collect, even for the purposes of harm reduction [15]. When criticisms emerge from outside of academia, they tend to involve social media, blog posts, and occasional *extreme* reactions, such as threats of violence or demands that a particular scholar be terminated [16]. In fact, the emergence of social media has fundamentally changed how such controversies play out, with new potential for “huge waves of outrage” to emerge rapidly in an “online firestorm” [17].

Rather than stemming from fundamentally irrational behavior, online firestorms often manifest as aggressive forms of *sousveillance* [18] that “use the available masses of weak ties in social media to publicly enforce social-political norms” [19]. While similarities have been drawn between firestorms and moral panics, a recent study suggested that “social appropriateness of attacking the denounced actor” was an important antecedent of firestorm participation, as opposed to a predetermined moral stance [20]. In fact, the *piling on* behavior observed in firestorms is a fundamental component of moral grandstanding—wanting to be perceived as moral by others [21]. Although firestorms may not promote long-standing change in public discourse [22], they can result in significant short-term consequences. This can be observed, for example, in the case of Justine Sacco, a communications executive who was publicly terminated after a firestorm erupted in response to a tweet she sent in 2014 [23]. These consequences can even accrue when the firestorm is based on misleading or false information, as with Dominique Moran who was fired in response to social media outrage accusing her of racism, though she was offered her job back after the full spectrum of evidence was presented [24].

### The Firestorm

In 2015, an annual ATOD survey was administered by local school officials to students in grades 6 through 12 in several hundred schools in a midwestern state. Participation in the survey by any school was voluntary. Schools that elected to participate were told that they had the choice of either electronic or paper administration formats; they were informed of their responsibility to seek either active or passive consent from parents and guardians for their children to participate in the survey. More than 25% of all schools in the state participated, and the aggregate student body of participating schools was predominantly white (approximately 70%), generally reflecting the state population.

As part of this process, the finalized survey instrument was made fully and openly available on a website for parental review for several months prior to survey administration, and schools were instructed to direct parents to that website. Schools were also instructed that at the time of survey administration, students should be told that completing the survey, as well as responding to individual survey items, was voluntary, even if their parent or guardian consented to their participation. At the beginning of the survey instrument, participants were prompted to indicate their date of birth, the initials of their first and last names, and the first letter of the school they attended at the beginning of the first grade. These data were to have been used to generate a confidential survey ID for the purposes of matching data between years, but were not designed to identify individuals, nor did the survey administrators have the ability to do so. This was the first year that this long-standing survey attempted to ask these questions, the purpose of which was to support confidential longitudinal data analyses. This information would have been used, for example, in identifying a particular grade or *cohort* with unique risks, which is commonly done in many areas of public health, for example, in Willets, 2004 [25]. Once the pilot test was fielded, the item asking for date of birth and initials raised parental curiosity. One of the families contacted a traditional media outlet, which aired a local television story about the survey.

The focus of the news story was not the new questions, though that was what had sparked interest. Instead, parents were mostly upset with several standardized risk and protective factor questions from the family domain of the Communities that Care (CTC) Youth Survey [26] having to do with “arguing about the same things in my family over and over,” “having serious arguments,” and “often insulting or yelling at each other.” Immediately following this story, the new questions, but not the CTC items, were removed from the final survey tool. Despite this, a minor firestorm erupted on social media in the comments section of the local news station’s page and a vitriolic debate ensued.

### This Study

School-based surveys support harm reduction and substance use prevention efforts. The concomitant risk of public controversy and, more recently, of a digital firestorm may make school officials, epidemiologists, and public health scholars wary of advancing this important work. To better understand the nature of digital firestorms targeting school-based ATOD surveys, this study analyzes the qualitative comments made on social media during a small firestorm targeting a school-based ATOD survey administered by an independent organization and funded by a state-level government agency. The study also uses that information to make recommendations for how those who administer school-based surveys might proactively set in place protective structures that minimize the likelihood of controversy.

## Methods

### Data Collection

Data were captured by archiving all public comments posted to social media in response to the news story described above (ie, *the firestorm*) about a school-based public health survey. Local media featured this story on television then published the story—the only story covering this topic—to their website and two social media outlets. On one of the social media platforms, the post had no active engagement of any type. Thus, study data were drawn exclusively from the other social media platform. The thread was active (ie, the time from posting the story to social media until the final comment was posted) for approximately 35 hours. Because of the nature of online debate, at the point of archiving, several of the more incendiary posts had either been made private or had been redacted prior to capture and were, therefore, not included in the dataset. Although all data were posted in a digital public forum, to protect individuals’ privacy, this study did not identify the social media platform in question; quotes that might potentially be used to identify individuals were paraphrased or edited for publication, without changing the tone or meaning, to remove colloquialisms that would render the strings unique.

This work was granted *Exempt* status by the Indiana University Institutional Review Board (number: 1511842027).

### Theoretical Orientation

More than a decade ago, researchers identified a “crisis of trust” in how the public at-large views science [27]. As lay exploration of science related to all topics, including public health, has moved to the Internet, it is probable that the level and type of interactivity matters in how content is understood and the ways that the public reacts to the content [28]. Further, comment sections in online science articles often veer from the initial story content, particularly in the direction of moral and ethical claims and community-driven narrative [29].

Because these comments were made in a public digital forum, they offered a naturalistic opportunity to explore and categorize the components of a digital firestorm targeting a public health survey tool. For this reason, we used a general inductive approach [30] informed by theoretical thematic analysis [31] to extract individual and overlapping categories from individual comments.

### Data Analysis

Data were transcribed into Dedoose and cleaned by the lead author (RAG), who then segmented comments into two broad categories: oppositional and supportive. For comments that were brief or unclear (eg, only the word “Agreed”), opposition or support was determined by context. Comments by the same person that were broken into multiple *posts* on the thread were counted as single *comments* when they focused on the same message, though this occurred very infrequently since this was a small firestorm.

A second reviewer validated comment sorting before the study moved forward. Then, within each of the two categories, the second researcher (1) completed multiple *deep reads* of the dataset, (2) generated preliminary themes and prepared a codebook, (3) assigned one or more codes to each social media comment, (4) revised the themes, and (5) recoded each individual social media comment. The lead author then (6) received the coded data and codebook, (7) reviewed each individual comment, (8) suggested revisions to the codebook, and (9) recoded each comment, highlighting any potential areas of disagreement. Finally, the two researchers met in person to discuss the data and continued shared analysis until 100% agreement on coding for each comment was achieved.

## Results

### Negative or Oppositional Comments

#### Overview

A total of 133 individual comments were coded as negative or oppositional. Within this segment, researchers identified seven categories, as outlined in Table 1.

The majority of comments (105/133, 78.9%) fell within a single categorical element. The most frequently shared categories were *government related* + *conspiratorial or irrational* (10/133, 7.5%) and *reasoned disagreement* + *survey mechanisms* (6/133, 4.5%). Most comments were easy to parse for categorical content, though a few posed conceptual difficulty and required lengthier discussion. For example, we coded a comment about children belonging to the state, and the potential for *resistance*, as *government related* and *conspiratorial or irrational*, but cases could be made for additional categories depending on how the intention of the comment is interpreted.

Frequencies and percentages of categories are provided in Table 2.

**Table 1:** Negative or oppositional comments.

Comment category	Description
Government related	This code was used to indicate comments referencing government overreach, as distinct from school overreach; criticisms of government in any form; or references to a political agenda. It specifically excluded conspiratorial thinking.
Conspiratorial or irrational	This code was used to indicate comments displaying conspiratorial thinking or creation of implications that demonstrably surpass a *reasonable person* standard (eg, comparison of a survey to actions taken by Nazi Germany). Importantly, simply expressing concern about the unique identifier did not qualify a comment for this code.
Parental autonomy and privacy	This code was used to indicate comments focusing on the roles or rights of parents, especially vis a vis the school; the appropriate role of the school versus other entities; or criticism of the school’s involvement in students’ home lives, including privacy concerns.
Child protective services and police	This code was used to indicate comments suggesting that the survey was a tool that could result in a law enforcement agency investigating parents.
Reasoned disagreement	This code was used to indicate criticism of the survey that attempted to make a logical case against it.
Survey mechanisms	This code was used to indicate concerns about survey mechanisms, including perceptions about informed consent, confidentiality, and the voluntary nature of its completion.
Other or miscellaneous	This code was used to categorize trolling within negative threads (eg, spelling correction), affirmation of negativity (eg, simply writing “Agreed”), or other outlier comments.

**Table 2:** Amount of negative or oppositional social media comments containing identified categorical elements.

Comment category^a^	Number of comments (N=133), n (%)
Government related	22 (16.5)
Conspiratorial or irrational	19 (14.3)
Parental autonomy and privacy	53 (39.8)
Child protective services and police	7 (5.3)
Reasoned disagreement	16 (12.0)
Survey mechanisms	15 (11.3)
Other or miscellaneous	33 (24.8)

^a^Categories were not mutually exclusive. The most common overlapping codes were *government related* + *conspiratorial or irrational* (10/133, 7.5%) and *reasoned disagreement* + *survey mechanisms* (6/133, 4.5%).

#### Government Related

Most respondents who discussed the government used this firestorm as a place to insert specific criticisms of the state or federal government, sometimes without any direct mention of the survey.

The state can’t accomplish anything positive...

Participant #4

That’s the public government indoctrination school system...

Participant #177

Other respondents enmeshed criticism or concern about the government with other thematic areas, including conspiratorial thinking and parental issues. Some participants argued that the survey was an attempt to violate civil rights and control children’s upbringing while *pretending* to be a process intended to protect youth. Others simply asserted government intrusiveness.

Government is...increasingly intrusive on people’s lives...

Participant #62

#### Conspiratorial or Irrational

Nearly all responses fitting this category referenced Nazi ideology or fascism, perhaps in loose observance of Godwin’s law [32]. One participant, for example, referenced Nazi Germany and then indicated that this type of questionnaire historically has been a tool used against parents by oppressive governments. Other responses used memes or articulated more specific, if unrealistic, concerns related to large-scale arrests of those whose children responded to the surveys in certain ways.

[Meme used as part of response: A picture of Anne Frank saying, “hiding away up in the attic, are we Anne Frank? Why don’t you just obey the law?”]

Participant #0

Soon they will [ask] children [whether their parents criticize] the school board...or other elected officials. Then [the parents will be sent] to jail just like...dictatorships [in the past]...

Participant #62

#### Parental Autonomy and Privacy

A number of respondents focused on defining what the school’s role should be in society, both in general and in distinction to other organizations or groups.

It’s not their place to ask!

Participant #201

That looks like a doctor’s office survey so leave it [there].

Participant #192

Other responses indicated a feeling of having their privacy invaded by the school, and implied that the schools were on a slippery slope in terms of the questions they were asking. One participant, for example, began listing sex positions and referenced the Kama Sutra in the context of stating that none of those things are anyone’s business, though no questions on the survey pertained to sexuality. Comments also sometimes suggested that this was an attempt for schools to gain authority over youth.

We need to take back control of our children...

Participant #1

What goes on in the home [isn’t] their business...schools already overreach...

Participant #19

#### Child Protective Services and Police

There was a small cluster of responses that specifically expressed concern that the survey would trigger legal action to seize children.

...my fear is [the survey] may also have [the government and child protective services]...causing trouble where none exists by taking answers out of context...

Participant #52

#### Reasoned Disagreement

Some respondents did not support or agree with the survey being conducted, but they approached their concerns in a measured manner. One participant suggested calmly instructing youth first to decline participation and then to notify the parents if they received pushback against that decision. Others posed theoretical disagreement or questions about the survey.

If the school [were concerned], they [wouldn’t necessarily have to ask] all of the kids [whether their parents argue all of the time].

Participant #67

#### Survey Mechanisms

A number of comments indicated concerns with the survey protocol, though it is not possible to tell whether these reflect actual lapses in protocol at individual schools or respondent concerns that are not based on actual events.

...students at <redacted> were not informed participation was voluntary, and were told they had to fill out the survey...

Participant #91

### Positive or Supportive Comments

#### Overview

A total of 74 individual comments were coded as positive or supportive. Within this segment, researchers identified six categories, as discussed in Table 3.

The majority of comments (48/74, 65%) fell within a single categorical element. The most frequently shared categories were *prevent abuse or neglect* + *hiding something* (8/74, 11%) and *factual statements* + *reasoned support* (7/74, 9%).

Frequencies and percentages of categories are provided in Table 4.

**Table 3:** Positive or supportive comments.

Comment category	Description
Prevent abuse or neglect	This code was used to indicate comments suggesting that the survey would serve to prevent or intervene with specific cases of abuse or neglect.
Holistic health	This code was used to indicate comments suggesting that youth are affected by home life or environment or justifying the school’s role in supporting home life.
Hiding something	This code was used to indicate comments suggesting that the only reason to be upset with youth completing the survey is that the parents have something to hide.
Factual statements	This code was used to indicate comments demonstrating a factual understanding of how the ATOD^a^ survey works.
Reasoned support	This code was used to indicate support for the survey that attempted to make a logical case for it.
Other or miscellaneous	This code was used to categorize trolling within supportive threads (eg, spelling correction), affirmation of positivity (eg, simply writing “Agreed”), or other outlier comments.

^a^ATOD: alcohol, tobacco, and other drug.

**Table 4:** Amount of positive or supportive social media comments containing identified categorical elements.

Comment category^a^	Number of comments (N=74), n (%)
Prevent abuse or neglect	17 (23)
Holistic health	17 (23)
Hiding something	18 (24)
Factual statements	15 (20)
Reasoned support	16 (22)
Other or miscellaneous	23 (31)

^a^Categories were not mutually exclusive. The most common overlapping codes were *prevent abuse or neglect* + *hiding something* (8/74, 11%) and *factual statements* + *reasoned support* (7/74, 9%).

**Prevent Abuse or Neglect**

A number of participants supported the survey because they believed that it could be used to identify specific youth who were experiencing adverse events (eg, abuse) and, in some cases, to intervene with those individuals.

...it is important for the truth to [be revealed so that youth can be saved]. I would be happy to [clarify my own actions with child protective services in order to help protect] other kids that are being abused or neglected...

Participant #51

Who had a problem, abusive parents?

Participant #99

#### Holistic Health

Other participants justified administration of the survey by asserting that home life often affects educational performance or that parents cannot or should not be inconsistent in requests that the school invest in their children’s lives.

[Anyone who says that home life has] nothing to do with education must be joking!

Participant #24

...You want the school to be involved [and not involved at the same time]. [Parents need to] figure this out...[it’s] the first step to ensure kids’ safety...

Participant #74

#### Hiding Something

Some individuals indicated that there was nothing to worry about as long as others had nothing to hide; others took this concept one step further, asserting that those who were upset about the survey specifically had something to hide from authorities or school personnel.

If there is nothing to hide it should not matter...

Participant #60

...[if you don’t want your own child to answer these questions], is it because you are scared of what they will say...? If you answered yes to either [of the questions about home life], you are the reason they have to ask these questions.

Participant #65

#### Factual Statements

A few participants provided statements outlining factual information about the ATOD survey. This included both comments related to the mechanics of survey administration as well as statements related to the implications of the survey (eg, what is intended to be done with the data).

The survey results are [used] to gather data...when you see stories that [describe the prevalence of youth smoking or drug use]...This is where that information comes from.

Participant #68

These surveys are given to every student...They are completely anonymous. Teachers [cannot] administer them [to preserve privacy].

Participant #30

#### Reasoned Support

Although there was a meaningful amount of overlap between factual understanding of the survey and reasoned support, there were also other supportive statements from individuals who did not assert knowledge about the survey but who attempted to reason through the debate.

[It would be hard to find a family that could honestly say they didn’t argue]...That’s part of family life...we don’t always say warm and fuzzy things...but [we] still [care for one another].

Participant #61

Schools...cannot win. [It’s unfortunate]...[what if we could potentially] offer support and programs [to] guardians who may need assistance?

Participant #82

## Discussion

### Principal Findings

This study reviewed the full spectrum of social media comments posted as part of a localized social media firestorm surrounding administration of a school-based ATOD survey. As one might expect, such discourse is primed for a certain amount of controversy [33]. Analyses found that the responses were split at the broadest levels of *opposition* or *support*, though nearly two-thirds (133/207, 64.3%) opposed or criticized the survey. Further, most comments focused on the survey itself, the perceived context in which it was offered, or broader claims about society; few responses displayed individually directed *flaming* behavior (eg, “negative violations of...interactional norms”) [34].

Notably, only 7.2% (15/207) of the total responses contained factual information about the ATOD survey, and many of the other comments specifically relied on inaccurate assumptions about the survey. It is objectively true that the survey responses could not have been linked to individual students by the survey administrators; however, many commenters who wrote oppositional messages, especially those related to child protective services and privacy, either were unaware of this fact or did not believe it to be true. Likewise, many conspiratorial or seemingly irrational comments presupposed a broader, sinister agenda, components of which would require the ability to link survey data to individual students. At the same time, it is important to avoid “white hat bias” [35] by noting that some comments supportive of the survey were also advanced based on the same incorrect premises; in particular, supportive statements suggested that the ATOD survey could prevent individual cases of abuse or maltreatment or that parents only disliked the CTC items because they might be caught hiding harmful behavior; however, neither of those scenarios were possible.

In total, many (99/207, 47.8%) coded comments were in the following categories: *parental autonomy and privacy*, *child protective services and police*, *conspiratorial or irrational*, *prevent abuse and neglect*, or *hiding something*. Although there was no overarching code for misinformation, comments in each of these categories, both positive and negative, logically presupposed inaccurate information about the purpose of the survey or the survey methods. In the context of digital moral outrage [36], one interpretation of the high volume of this type of comment is that the negative comments were indicating that the survey’s perceived invasions of privacy or question content violated social or moral norms in varying ways. On the other hand, the positive comments were presenting oppositional norms in which child safety was more highly valued than privacy or preferences about the kinds of questions that should be posed to youth.

Consistent with prior research [29], some of the comments not directly related to privacy, both positive and negative, also relied on a priori personal or community-based normative beliefs. This was most commonly observed in the *government related* and *holistic health* categories, manifesting as general mistrust or disdain for the government in the former and an underlying belief or overt assertion that home life is interlinked with school life in the latter. Many comments in these categories could have been posted as template responses to any news story focusing on government activity or the role of the school in raising youth.

One important general finding from this study is that while this social media firestorm occurred in the context of a school-based ATOD survey, much of the online activity was driven by factors external to the survey itself, though the consequences to the survey administration process could easily have been substantial. In fact, organizations administering school-based ATOD surveys have a vested interest in avoiding controversy, especially in online formats. Based on the data from this study, it is tempting simply to suggest that more specific, directed information provision about the nature of the survey and the purpose of administration would serve as *firestorm protection* for school-based ATOD surveys. This is an especially compelling argument given that this particular firestorm included a high density of conflict (ie, prosurvey and antisurvey) stemming from norms unrelated to the actual survey itself. At the same time, such an approach likely must be nuanced and should not minimize or trivialize these norms. For example, if there is widespread pre-existing concern about privacy, in general, in a segment of the population, then it may be beneficial to avoid the appearance of violating privacy, even when such a thing could not occur in practice.

### Recommendations

The data in this study cannot determine whether misunderstandings about the survey stemmed from a lack of information availability or from a lack of belief in available information. It is plausible that the reality is a mixture of the two. Researchers have long acknowledged that “public understanding of science, and of public risk perceptions, are not so much about public capabilities...but about the trust and credibility they are prepared to invest...” [37]. This may extend beyond conspiratorial or government-related concerns to the schools themselves, which may not be trusted [38] or which may, as seen in this study, be viewed solely as an adversarial government apparatus. Thus, while it may be necessary to thoroughly outline survey procedures and protocols for public consumption, it is likely not sufficient. This study also suggests the importance of providing clarity about the survey’s purpose, how the data will be used, how they will be safeguarded, and who oversees the survey at each level. Further, there may be value in facilitating some level of community member coconstruction of the messaging, both to engender trust and to identify potential *pain points* that might spark outrage [39].

There is also a documented disconnection between public outrage and objective level of risk, especially when risks are perceived as involuntary or unfamiliar [40]. It is important to acknowledge the community’s perceived risks associated with data collection, even when they may objectively be minimal, as meaningful concerns [41] and to take additional steps to mitigate or address them. The most obvious step is to avoid collecting anything that might appear to link data to participants, even when safeguards are in place to prevent that from occurring. Other strategies might also include additional or redundant layers of student protection, for example, messaging written on the survey itself indicating, “even if you have been instructed by your teacher that you must take this survey, you are not required to do so.”

Ultimately, however, prior research [15,18-21,29] suggests that online firestorms are complex and often emerge as a result of many factors outside of the context of the specific *target*. Data from this study do nothing to attenuate that claim, with a significant digital presence in the firestorm allocated to a priori beliefs about the government, child welfare, and society. Thus, it is likely impossible to be 100% protected against the possibility of digital outrage when conducting a school-based survey. At the same time, depending on the scope or intent of a given public health project (eg, global immunization), it may be possible to generate targeted social media messaging to assuage some concerns (eg, the World Health Organization’s syndicated, organized, social media campaigns) [42].

### Limitations and Study Context

This study is subject to several limitations. First, although all data points were captured and reviewed, this study was intended to deeply understand this particular incident. One cannot assume that all digital firestorms will include similar content, though we suggest applicability of these findings specifically to school-based ATOD surveys administered for epidemiological purposes. Second, the study team did not compute interrater reliability for the coding, choosing instead to complete iterative review until 100% concordance was reached. Interrater reliability for qualitative research has long been a subject of discussion among scholars, who have noted that agreement on basic themes can easily be achieved, but interpretation of those themes can diverge [43]. Such divergence has been observed even in highly rigorous qualitative studies and tends to result from complex information (ie, multiple themes and subthemes) within a single piece of data [44]. Because of the nature of the data in this study—generally short, written statements rather than transcripts of interviews or focus groups and the a priori determination of the research interest (ie, the survey)—it was feasible to achieve full agreement on themes and interpretation relative to the research topic among the coders. Finally, this study did not have the ability to determine whether comments originated from people in the state where the firestorm occurred, nor was it able to determine whether comments were from online trolls or social bots intending to influence the discussion, for example, in Ferrara et al, 2016 [45].

To the authors’ knowledge, there are no extant studies that have investigated digital outrage relative to public health ATOD surveys in schools and few studies that have researched this in public health in general. Much of the writing about online firestorms has examined marketing and branding, including stakeholder relations and online *paracrisis* (ie, “a publicly visible crisis threat that charges an organization with irresponsible or unethical behavior”) [46]. This work has included, for example, experimental assessment of a proposed model of paracrisis development in response to a visual ad [47] and nonacademic books designed to help businesses (eg, *How to Protect [or Destroy] Your Reputation Online*) [48]. This study advanced understanding of the online firestorm phenomenon specifically as it pertains to school-based epidemiological surveys, which was heretofore mostly unexplored. At the same time, as noted above, it is limited in scope to the specific incident described and does not address, nor was it intended to address, the more general research literature conceptualizing digital outrage.

## Abbreviations

ATOD: alcohol, tobacco, and other drug
CTC: Communities that Care
